# The chemokine fractalkine (CX3CL1) attenuates H_2_O_2_-induced demyelination in cerebellar slices

**DOI:** 10.1186/s12974-017-0932-4

**Published:** 2017-08-15

**Authors:** Sinead A. O’Sullivan, Kumlesh K. Dev

**Affiliations:** Drug Development, School of Medicine, Trinity College Dublin, Dublin, Ireland

**Keywords:** Fractalkine, CX3CL1, GOX-CAT, Hydrogen peroxide, demyelination

## Abstract

**Background:**

Fractalkine/CX3CR1 signalling has been implicated in many neurodegenerative and neurological diseases of the central nervous system (CNS). This signalling pathway plays an important role in regulating reactive oxygen species (ROS), as well as itself being altered in conditions of oxidative stress. Here, we investigated the effects of recombinant fractalkine (rCX3CL1) in models of hydrogen peroxide (H_2_O_2_)-induced demyelination and astrocyte toxicity, within organotypic cerebellar slice cultures.

**Methods:**

Organotypic cerebellar slice cultures were generated from postnatal day 10 C57BL/6J mice to assess myelination. Immunohistochemistry was used to measure the degree of myelination. Fluorescent images were obtained using a leica SP8 confocal microscope and data analysed using ImageJ software.

**Results:**

We show here, for the first time, that rCX3CL1 significantly attenuated bolus H_2_O_2_-induced demyelination as measured by expression of myelin basic protein (MBP) and attenuated reduced vimentin expression. Using the GOX-CAT system to continuously generate low levels of H_2_O_2_ and induce demyelination, we observed similar protective effects of rCX3CL1 on MBP and MOG fluorescence, although in this model, the decrease in vimentin expression was not altered.

**Conclusions:**

This data indicates possible protective effects of fractalkine signalling in oxidative stress-induced demyelination in the central nervous system. This opens up the possibility of fractalkine receptor (CX3CR1) modulation as a potential new target for protecting against oxidative stress-induced demyelination in both inflammatory and non-inflammatory nervous system disorders.

## Background

Fractalkine (CX3CL1) and its receptor, fractalkine receptor (CX3CR1), are constitutively expressed in the central nervous system. This is in contrast to most other chemokines, whose expression in the brain is only detected during inflammation [1–3]. One of the unique features of fractalkine is its ability to exist as both a membrane-tethered adhesion molecule and as a soluble chemotactic ligand [4]. In naive rodent brains, CX3CR1 has been localised to microglia [1, 5] and shown to be expressed intracellularly by neurons [5]. Fractalkine is shown to be localised to neurons [1, 5] and astrocytes [5, 6]. It is thought that fractalkine expression in the brain is an important mediator of neuronal-glial communication. It is also thought to play a homeostatic role in normal physiological functions with the ability to regulate immune responses during times of injury [7, 8].

Several studies provide evidence of neurotoxic insults increasing the expression of fractalkine on neurons and astrocytes as well as increased microglial migration [9–11]. In the rodent experimental autoimmune encephalomyelitis (EAE) model for multiple sclerosis (MS), an increase in astrocytic levels of fractalkine was observed at sites of inflammation, while neuronal fractalkine remained unchanged. Microglial CX3CR1 expression has also been reported to be increased around active demyelinating lesions [12]. Further experiments suggest that fractalkine plays a direct neuroprotective role in the regulation of inflammation under oxidative and ischemic conditions [13, 14]. Fractalkine has also been shown to promote human monocyte survival through a reduction in intracellular reactive oxygen species (ROS) [15]. However, studies also show that ROS such as hydrogen peroxide (H_2_O_2_) can enhance the expression of fractalkine and other adhesion molecules on endothelial cells, which may contribute to vascular injury [16]. Thus, evidence suggests that fractalkine can be neuroprotective or neurotoxic depending on the cellular environment [17–19].

Ischemic brain injury has been associated with the generation of excess ROS, which can lead to oxidative stress and, in turn, neuronal damage [20]. Increases in ROS production have also been shown to affect myelin-producing oligodendrocytes. High concentrations of iron and polyunsaturated fatty acids combined with low levels of glutathione make oligodendrocytes an ideal target for oxidative stress [21]. Decreasing the levels of ROS surrounding these oligodendrocytes has been shown to increase myelin production with a concurrent reduction in the anti-oxidative response elements Nrf2 and hemeoxygenase-1 (HO-1) [21, 22].

Earlier work in our lab has investigated fractalkine signalling as well as the effects of glucose oxidase-catalase (GOX-CAT) and bolus H_2_O_2_ on cell viability of isolated astrocyte cultures [6, 23]. We have also established previously a model of GOX-CAT-induced demyelination [23] as well as bolus H_2_O_2_ to compare low-continuous versus high-acute levels of oxidative stress in organotypic cerebellar slice cultures. In these studies, we have shown that GOX-CAT and bolus H_2_O_2_ can cause demyelination, which is attenuated by the MS drug, FTY720/Gilenya [23]. Due to documented protective effects of fractalkine in oxidative stress conditions [13–15] and the particular susceptibility of oligodendrocytes to oxidative stress [21], here, we build on these previous works and investigated the protective effects of recombinant fractalkine in an organotypic slice culture system investigating myelination under oxidative stress conditions.

## Methods

### Compounds and treatments

Recombinant mouse CX3CL1/fractalkine chemokine domain (R&D systems; 458-MF) was used at various concentrations as indicated in the figure legends. Hydrogen peroxide (H_2_O_2_; Sigma, 216763) was prepared fresh for every experiment by diluting appropriately in serum-free media. Glucose oxidase solution (GOX; G0543) and catalase solution (CAT; C3155) were both sourced from Sigma. GOX was supplied at a concentration of 200 U/mg, where 1 unit of enzyme activity oxidises 1 μM of d-glucose per minute to H_2_O_2_. GOX was used at a fixed concentration of 0.1 units/ml (i.e. 1:100,000 dilution) throughout all experiments. CAT was supplied at a concentration of 30,000 U/mg, where 1 unit of enzyme activity decomposes 1 μM of H_2_O_2_ per minute to H_2_O. CAT was used at varying concentrations from 0.03 units/ml (i.e. 1:1,000,000 dilution) to 300 units/ml (i.e. 1:100 dilution) as indicated in the figure legends. All GOX-CAT treatments were allowed to equilibrate for approximately 15 min prior to addition to slice cultures.

### Mouse organotypic cerebellar slice culture

Cerebellar slice cultures were prepared as described previously [23–27] using brain tissue isolated from both male and female postnatal day 10 C57BL/6J mice, according to the protocol reported earlier [26]. Mice were decapitated; cerebellar tissue was removed from the skull and separated from the hindbrain with spatulas on ice. The cerebellum was cut into 400-μm parasagittal slices using a McIlwain tissue chopper. Slices were separated into individual slices under a dissection microscope. Four slices were grown on each cell culture insert (Millicell, PICMORG50). Slices were cultured in media containing 50% Opti-MEM (Invitrogen, 11058021), 25% Hanks’ buffered salt solution (HBSS, Invitrogen, 14025-050), 25% heat inactivated horse serum (Biosera, HO-290) supplemented with 2 mM Glutamax (Invitrogen, 35050-038), 28 mM d-glucose (Sigma, G8769), 1% pen/strep, and 10 mM HEPES (Sigma, H3784) for 12–14 days in vitro. Slice cultures were grown at 35.5 °C and 5% CO_2_ in a humidified incubator. Slices were starved in serum-free media for 4 h prior to all treatments.

### Immunohistochemistry

Immunohistochemistry for organotypic slices was performed by washing the slices twice in PBS. The slices were then fixed by incubation in 4% formaldehyde solution (Sigma, F1635) for 10 min. The slices were washed twice in PBS and incubated overnight in blocking buffer (PBS supplemented with 10% BSA and 0.5% Triton X-100). For all antibody dilutions, PBS supplemented with 2% BSA and 0.1% Triton-X 100 was used. Slices were incubated for 24 h at 4 °C in primary antibody, washed three times in PBS supplemented with 0.01% Triton X-100 and incubated overnight at 4 °C in secondary antibody. The slices were washed again three times and mounted on glass cover slides in antifade reagent (life technologies, S36936) and the edges sealed with varnish. Samples were stored at 4 °C in the dark until imaged. Slices were visualised with a Leica SP8 confocal microscope at × 10 magnifications. The primary antibodies used were myelin basic protein (MBP) (Abcam, ab40390), myelin oligodendrocyte glycoprotein (MOG, Millipore; MAB5680), neurofilament heavy (NFH, Millipore; ab5539) and vimentin (Santa Cruz, sc373717). Secondary fluorescent antibodies used were goat anti-rabbit Alexa 488 (Invitrogen, A11008), goat anti-chicken Alexa 633 (Invitrogen A21103) and anti-mouse Dylight 549 (Jackson Immunoresearch, 115-506-068). Four slices per condition were grown and six to eight images per slice were taken to cover the whole slice, thereby limiting bias and variation between conditions. Values in graphs represent the mean fluorescent intensity of approximately 130 measurements with standard error of the mean. Confocal images were captured as 12-bit.lif files of 1024 × 1024 pixel resolution. Image acquisition settings were kept constant across treatments. Image analysis was conducted using ImageJ software (https://imagej.nih.gov/ij/).

### Statistical analysis

Using ImageJ, confocal images were split into single channels and a minimum threshold was adjusted to remove background staining/noise. Arbitrary fluorescence values were averaged across the four slices per condition to give a single value for the total fluorescent intensity of that condition. All statistical analysis was performed using GraphPad Prism 5. In experiments where three or more groups were compared, an ordinary one-way analysis of variance (ANOVA) was performed and was followed by post hoc tests. Tukey’s post hoc test was used for experiments where all columns were compared to each other. Student’s *t* test was used to compare the means between two groups. Detailed data analysis methods are provided in the figure legends and ‘Results’ sections.

## Results

### Fractalkine (CX3CL1) prevents bolus H_2_O_2_-induced demyelination in cerebellar slices

**Fig. 1:**
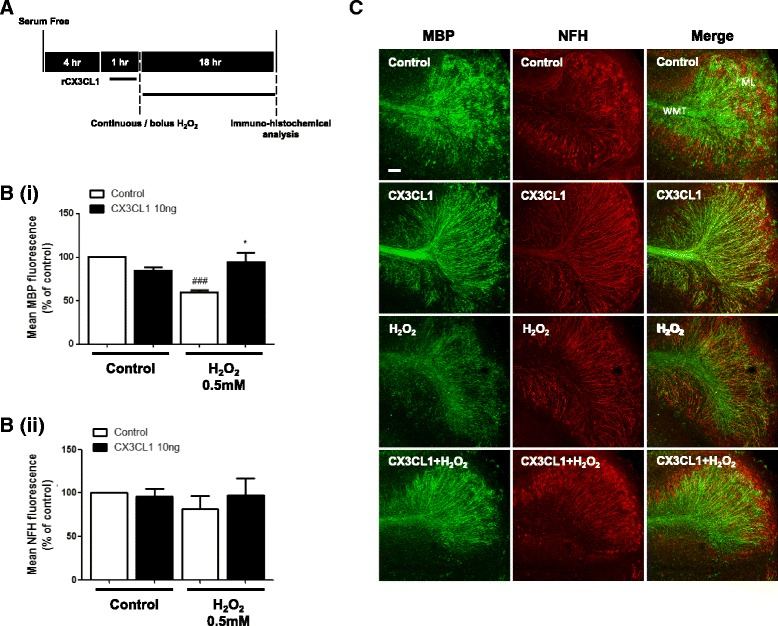
Fractalkine prevents bolus H_2_O_2_-induced demyelination in cerebellar slice cultures. **a** Experimental timeline shown. **b(i)** Organotypic cerebellar slices were pre-treated with fractalkine (CX3CL1; 10 ng) for 1 h prior to addition of bolus H_2_O_2_ (0.5 mM) for 18 h. Graph shows a significant decrease in MBP fluorescence after H_2_O_2_ treatment. This effect is rescued with CX3CL1 treatment. **b(ii)** NFH shows no significant decrease with bolus H_2_O_2_ treatment compared to control. **c** Images are representative of three separate experiments. Confocal images taken at × 10 magnification. *Scale bar*, 100 μm. Molecular layer (*ML*) and white matter tract (*WMT*) labelled. Data expressed as mean ± SEM; Student’s *t* test, ^###^ *p* < 0.001; and one-way ANOVA and Tukey’s post hoc test, *p < 0.05

Oxidative stress is thought to play a role in ageing as well as many neurodegenerative diseases [28]. Fractalkine and CX3CR1 expression has been shown to be altered in demyelinating lesions associated with EAE [12, 29]. To determine whether the soluble fractalkine ligand (sCX3CL1) is protective of myelin, in an environment of oxidative stress, we pre-treated cerebellar slices for 1 h with recombinant fractalkine (rCX3CL1; R&D systems; cat#458-MF) prior to addition of 0.5 mM bolus H_2_O_2_. Our results show that, after 18 h of bolus H_2_O_2_ treatment, there is a significant decrease in MBP fluorescence (59.6 ± 2.5%), which was significantly attenuated in groups pre-treated with rCX3CL1 (94.5 ± 10.3%) (Student’s *t* test, ^###^ *p* < 0.001, and one-way ANOVA and Tukey’s post hoc test, *p < 0.05) (Fig. 1B(i)). We also note a small decrease in NFH fluorescence in comparison to the control; however, this was not found to be significant (81.7 ± 14.6%) (Fig. 1B(ii)). This data shows that rCX3CL1 may have protective effects on myelin state in the cerebellum, in an environment of oxidative stress.

### Fractalkine (CX3CL1) prevents bolus H_2_O_2_-induced astrocyte cell death in cerebellar slice cultures

**Fig. 2:**
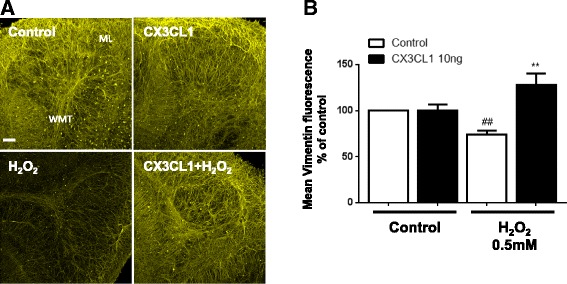
Fractalkine prevents bolus H_2_O_2_-induced astrocyte cell death in cerebellar slice cultures. Organotypic cerebellar slices were pre-treated with fractalkine (CX3CL1; 10 ng/ml) for 1 h prior to addition of bolus H_2_O_2_ (500 μM) for 18 h. **a** Images are representative of three separate experiments. Confocal images taken at × 10 magnification. *Scale bar*, 100 μm. Molecular layer (*ML*) and white matter tract (*WMT*) labelled. **b** Graph shows a significant decrease in vimentin fluorescence when treated with bolus H_2_O_2_. This effect is rescued when pre-treated with fractalkine. Data expressed as mean ± SEM; Student’s *t* test, ^##^ *p* < 0.01; and one-way ANOVA and Tukey’s post hoc test, **p < 0.01

CX3CL1 has been shown to have anti-apoptotic effects on human monocytes through a reduction in intracellular oxidative stress [15]. In certain diseases, the upregulation of CX3CL1 has been shown to help prevent microgliosis, through the activation of the NRF2 transcription factor and upregulation of heme oxygenase 1 proteins [30]. Therefore, in addition to the positive effects of rCX3CL1 on myelination in an organotypic slice model of oxidative stress, we next examined the effect of 0.5 mM bolus H_2_O_2_ on astrocytes. Vimentin fluorescence was significantly decreased through the addition of bolus H_2_O_2_ (74.2 ± 4.0%) (Fig. 2a, b). Importantly, pre-treatment with rCX3CL1 (10 ng/ml) significantly prevented this loss in vimentin fluorescence (127.7 ± 12.6%) (Student’s *t* test, ^##^ *p* < 0.01, and one-way ANOVA and Tukey’s post hoc test, **p < 0.01) (Fig. 2b). These results show that rCX3CL1 displays a significant protective effect on astrocytes in cerebellar tissue when exposed to large concentrations of H_2_O_2_.

### GOX-CAT-induced demyelination is prevented with fractalkine (CX3CL1) treatment

**Fig. 3:**
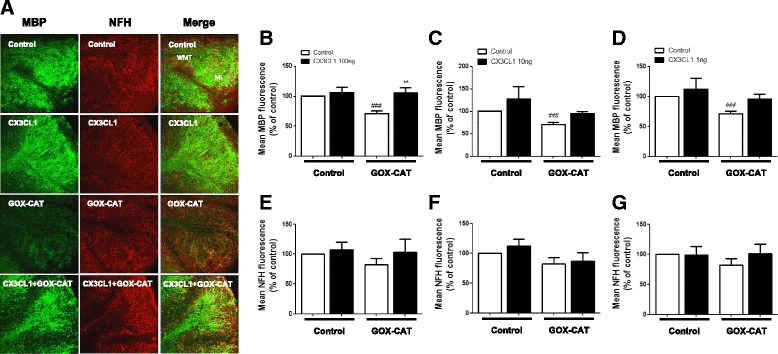
Fractalkine prevents oxidative stress-induced demyelination in cerebellar slice cultures. Organotypic cerebellar slices were pre-treated with fractalkine (*CX3CL1*; 100,10 or 1 ng/ml) for 1 h prior to treatment with glucose oxidase (*GOX*) and catalase (*CAT*); concentrations were maintained constant at 1:100,000 and 1:500,000, respectively, to generate low levels of H_2_O_2_ continuously. **a** Images are representative of fractalkine (CX3CL1) at 100 ng/ml treatment group. Confocal images taken at × 10 magnification. *Scale bar*, 100 μm. Molecular layer (*ML*) and white matter tract (*WMT*) labelled. **b** Graph shows a significant decrease in MBP fluorescence with GOX-CAT treatment. This effect is rescued with fractalkine treatment at 100 ng/ml but not at **c** 10 ng/ml or **d** 1 ng/ml. NFH shows no significant decrease in fluorescence with GOX-CAT treatment (**e**–**g**). Graphs expressed as mean ± SEM; Student’s *t* test, ^###^ *p* < 0.001; and one-way ANOVA, **p < 0.01; *n* = 5

Administration of large bolus doses of H_2_O_2_ is now being challenged as an inaccurate model of oxidative stress, largely because it involves non-physiological H_2_O_2_ concentrations [31]. Bolus H_2_O_2_ is also said to be metabolised within minutes [32]. Alternatively, the glucose oxidase-catalase (GOX-CAT) system is thought to produce physiologically appropriate concentrations of H_2_O_2_ over a physiologically appropriate time [23, 33]. Therefore, differing biological outcomes may result from either large bolus or low continuous H_2_O_2_ delivery. Given we find that rCX3CL1 protects myelin and astrocytes from the effects of a large bolus dose of H_2_O_2_ (Figs. 1 and 2), we next investigated if rCX3CL1 would have similar protective effects when using the GOX-CAT model. Cerebellar slices were treated with 100 ng/ml, 10 ng/ml or 1 ng/ml of rCX3CL1 for 1 h prior to treatment with GOX-CAT (GOX dilution 1:100,000, CAT dilution 1:500,000) for 18 h. This GOX-CAT treatment caused significant demyelination (70.8 ± 4.6%, Student’s *t* test, ^###^ *p* < 0.001; *n* = 4) (Fig. 3b–d), which was attenuated by pre-treatment for 1 h with rCX3CL1 (100 ng/ml) (105.4 ± 9.0%, one-way ANOVA and Tukey’s post hoc test, **p < 0.01; *n* = 4) (Fig. 3b). These effects of rCX3CL1 were concentration-dependent, where pre-treatment with 10 ng/ml (94.9 ± 4.7%) and 1 ng/ml of rCX3CL1 (95.8 ± 7.8%) did not have a significant protective effect (one-way ANOVA and Tukey’s post hoc test) (Fig. 3c, d). In the conditions tested, we observed no significant difference in NFH fluorescence (Fig. 3e–g). There were also no significant effects of rCX3CL1 treatment alone on MBP fluorescence, at any of the three concentrations tested (100 ng/ml, 105.8 ± 9.2%; 10 ng/ml, 127.3 ± 27.9%; 1 ng/ml, 112.0 ± 18.3%) and NFH fluorescence (100 ng/ml, 107.1 ± 12.7%; 10 ng/ml, 112.2 ± 11.2%; 1 ng/ml, 98.9 ± 13.9%) (Fig. 3e–g).

### GOX-CAT-induced reduction in MOG is attenuated by fractalkine

**Fig. 4:**
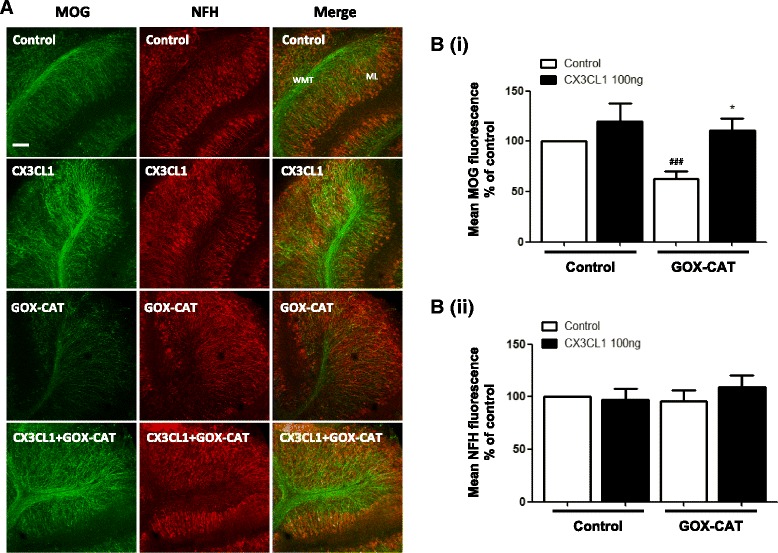
GOX-CAT-induced reduction in MOG is attenuated by fractalkine. Organotypic cerebellar slices were pre-treated with fractalkine (*CX3CL1*; 100 ng/ml) for 1 h prior to addition of glucose oxidase (*GOX*; 1:100,000) and catalase (*CAT*; 1:500,000). **a** Images are representative of five separate experiments. *Scale bar*, 100 μm. Molecular layer (*ML*) and white matter tract (*WMT*) labelled. Confocal images taken at × 10 magnification. **b(i)** Graph shows a significant decrease in MOG fluorescence with GOX-CAT treatment. This effect is rescued with fractalkine treatment at 100 ng/ml. **b(ii)** NFH shows no significant decrease in fluorescence with GOX-CAT treatment. Graphs expressed as mean ± SEM; Student’s *t* test, ^###^ *p* < 0.001; and one-way ANOVA, *p < 0.05; *n* = 5

In vitro studies on oxidative stress have led to mixed reports as to the protective [15] or toxic effects [16, 34] of fractalkine. Like many other cytokines, fractalkine has been shown to be either anti-inflammatory [35] or neurotoxic [36] depending on the circumstances. It has been suggested that the timing and concentration of fractalkine administration is of importance in determining the response of neurons and glia to potential neurodegeneration [37]. Given we find that soluble fractalkine attenuates GOX-CAT-induced decrease in MBP (Fig. 3), we further investigated the effects of GOX-CAT and recombinant fractalkine (rCX3CL1) on levels of MOG. We report that GOX-CAT treatment caused a significant decrease in MOG fluorescence compared to control (100 vs 62.7 ± 7.4%). Pre-treatment with rCX3CL1 (100 ng/ml) for 1 h produced significant protective effects on MOG levels (110.7 ± 11.9%, Student’s *t* test, ^###^ *p* < 0.001, and one-way ANOVA, *p < 0.05; *n* = 5) (Fig. 4B(i)). As before, we show that neither rCX3CL1 nor GOX-CAT significantly reduce NFH fluorescence (Fig. 4B(ii)). These finding suggest fractalkine can significantly attenuate the toxic effects of low continuous H_2_O_2_ on myelin proteins.

### Fractalkine does not protect astrocytes from low continuous H_2_O_2_ exposure

**Fig. 5:**
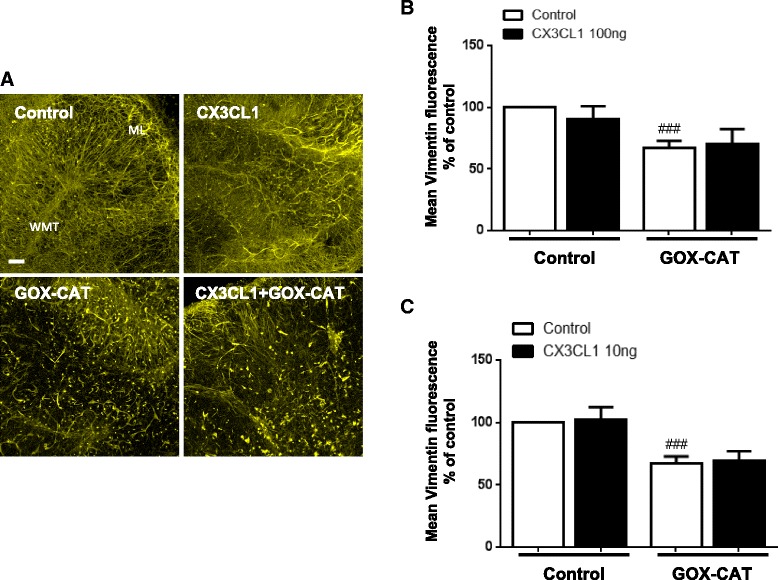
Fractalkine does not protect astrocytes from low continuous H_2_O_2_ exposure. Cerebellar slices were pre-treated with fractalkine (*CX3CL1*; 100 or 10 ng/ml) for 1 h prior to addition of glucose oxidase (*GOX*; 1:100,000) and catalase (*CAT*; 1:500,000) in order to generate low levels of H_2_O_2_ continuously. **a** Images are representative of five separate experiments. Confocal images taken at × 10 magnification. *Scale bar*, 100 μm. Molecular layer (*ML*) and white matter tract (*WMT*) labelled. **b**, **c** Graph shows a significant decrease in vimentin fluorescence when treated with GOX-CAT. Fractalkine treatment does not prevent this astrocyte cell death. Data expressed as mean ± SEM; Student’s *t* test, ^###^ *p* < 0.001; and one-way ANOVA with Tukey’s post hoc test

Many in vitro studies on the effects of oxidative stress on astrocytes have used large bolus H_2_O_2_ concentrations in order to measure biological effects [38–40]. In this study, we have used the GOX-CAT system to generate low continuous concentrations of H_2_O_2_ in order to induce demyelination in cerebellar slice cultures. In this model, fluorescence levels of the astrocyte marker vimentin decreased significantly when exposed to GOX-CAT (GOX dilution 1:100,000, CAT dilution 1:500,000) for 18 h (66.9 ± 5.9%) (Fig. 5b, c). Pre-treatment with rCX3CL1 for 1 h at 100 ng/ml (70.4 ± 11.8%) (Fig. 5b) and 10 ng/ml (69.6% +/− 7.4%) (Fig. 5c) (one-way ANOVA and Tukey’s post hoc test, *n* = 4) were unable to significantly attenuate this effect of GOX-CAT treatment on astrocytes. This data shows that even though rCX3CL1 is able to significantly attenuate the bolus H_2_O_2_-induced decrease in vimentin fluorescence (see Fig. 2), rCX3CL1 is unable to counteract the toxic effects of low continuous H_2_O_2_. These results may be explained by astrocytes displaying a higher vulnerability to continuous low-level concentrations of H_2_O_2_, in comparison to transient large bolus concentrations.

## Discussion

High concentrations of H_2_O_2_ can cause oxidation of proteins and lipids resulting in cellular damage [41]. To date, most studies have focused on bolus H_2_O_2_ treatments for studying the toxic effects of H_2_O_2_ on isolated cells. We and others have previously shown that addition of GOX-CAT enzymes allows for the generation of low-sustained levels of H_2_O_2_ in vitro, which may better mimic the sustained rise in oxidative stress levels seen in neurological disorders [23, 42, 43]. Given that a large amount of oxidative damage is associated with the initiation and progression of many neurological diseases, it is important to identify new cellular targets for preventing oxidative injury. Fractalkine is a chemokine which can regulate neuronal and glial cell responses to oxidative stress [14, 30]. Here, we investigated the effects of recombinant fractalkine (rCX3CL1) in cerebellar slices treated with bolus H_2_O_2_ or GOX-CAT-mediated H_2_O_2_. Addition of fractalkine to cerebellar slices, prior to H_2_O_2_-induced oxidative stress, significantly attenuates the loss of myelin associated with toxic levels of H_2_O_2_. Turning our attention to astrocytes, fractalkine significantly attenuates bolus H_2_O_2_-induced decrease in vimentin fluorescence but has little to no protective effect over GOX-CAT treatment. Overall, these studies suggest that fractalkine may protect astrocytes against highly concentrated but short lived H_2_O_2_ insults but may be limited in its protective effects against low-sustained levels of H_2_O_2_.

### Protective effects of fractalkine on myelination

The involvement of fractalkine in regulating the oxidative stress response has been documented. Administration of fractalkine prior to ischemic insult in wild-type rodents has been shown to produce neuroprotective effects, with smaller infarct sizes and a reduced mortality rate reported [19]. In contrast, studies with CX3CR1^−/−^ and CX3CL1^−/−^ rodents following ischemic injury had a better outcome in comparison to their wild-type counterparts which suggests that fractalkine contributes to neurotoxicity [18, 19]. This apparent contradiction highlights the complex signalling pathways evoked by fractalkine suggesting it can be protective or toxic depending on the microenvironment. In our studies, we show that H_2_O_2_-induced demyelination, as expressed by a decrease in the levels of MBP and MOG fluorescence, can be attenuated through the addition of fractalkine, prior to H_2_O_2_ insult. Of note, the fractalkine chemokine domain and not full-length fractalkine, which includes the mucin stalk, was used in this study. Addition of recombinant fractalkine (rCX3CL1) prior to H_2_O_2_ insult may be an important factor for the protective effects observed.

### Contrasting responses of astrocytes to bolus- and GOX-CAT-generated H_2_O_2_

Studies have suggested that different sub-populations of astrocytes in the brain can vary considerably in sensitivity to bolus H_2_O_2_. For example, hippocampal astrocytes are sensitive to concentrations as low as 50 μM, whereas cortical and cerebellar astrocytes are resistant to 500 μM H_2_O_2_ [44–46]. Astrocytes may also vary in their sensitivity towards the method of H_2_O_2_ delivery as it has been suggested that different or opposing biological outcomes may be elicited by cells in response to bolus or GOX-CAT H_2_O_2_ [31]. Using organotypic cerebellar slice cultures, decreased fluorescent intensity of vimentin, an intermediate filament protein in astrocytes, is caused by both bolus- and GOX-CAT-generated H_2_O_2_. In contrast to GOX-CAT-generated H_2_O_2_, rCX3CL1 significantly attenuated bolus H_2_O_2_-induced decrease in vimentin fluorescence. The protective effects of rCX3CL1 on bolus H_2_O_2_ insult may be due to the short half-life of H_2_O_2_ in cell culture [33, 40]. Similarly, the lack of protective effects observed in GOX-CAT-treated slices may be due to the half-life of rCX3CL1, which for chemokines is generally thought to be short [28, 47, 48]. In addition, persistent H_2_O_2_ signalling due to GOX-CAT activity, which is likely beyond the duration of rCX3CL1 signalling, outlasts any protective effects of rCX3CL1. Thus, rCX3CL1 may be protective of astrocytes in conditions of short-lasting bolus H_2_O_2_ treatments, but not in long-lasting continuous H_2_O_2_.

## Conclusions

In conclusion, we demonstrate that fractalkine signalling pathways may not be protective of vimentin within cerebellar slices from continuous, low concentrations of H_2_O_2_ whereas it has significant protective effects on vimentin when exposed to a once off, large bolus concentration of H_2_O_2_ as observed through an increase in vimentin fluorescence. We also show in our study that rCX3CL1 may protect myelin from pathological levels of H_2_O_2_ during oxidative stress conditions.

## Abbreviations

CNS: Central nervous system
CX3CL1: Fractalkine
CX3CR1: Fractalkine receptor
EAE: Experimental autoimmune encephalomyelitis
GOX-CAT: Glucose oxidase-catalase
H_2_O_2_: Hydrogen peroxide
HO-1: Hemeoxygenase-1
MBP: Myelin basic protein
ML: Molecular layer
MOG: Myelin oligodendrocyte glycoprotein
MS: Multiple sclerosis
NFH: Neurofilament heavy
rCX3CL1: Recombinant fractalkine
ROS: Reactive oxygen species
WMT: White matter tract
